# Improving Tanzanian childbirth service quality

**DOI:** 10.1108/IJHCQA-10-2015-0122

**Published:** 2018-04-16

**Authors:** Jennie Jaribu, Suzanne Penfold, Cathy Green, Fatuma Manzi, Joanna Schellenberg

**Affiliations:** 1 Epidemiology and Public Health, Swiss Tropical and Public Health Institute, Basel, Switzerland; 2 Department of Disease Control, London School of Hygiene and Tropical Medicine, London, UK; 3 Independent Advisor, Derbyshire, UK; 4 Ifakara Health Institute, Dar es Salaam, Tanzania; 5 Department of Disease Control and Department of Infectious Disease Epidemiology, London School of Hygiene and Tropical Medicine, London, UK

**Keywords:** Tanzania, Quality improvement, Birth plan, Health facility delivery, Partograph, Pregnancy danger signs

## Abstract

**Purpose:**

The purpose of this paper is to describe a quality improvement (QI) intervention in primary health facilities providing childbirth care in rural Southern Tanzania.

**Design/methodology/approach:**

A QI collaborative model involving district managers and health facility staff was piloted for 6 months in 4 health facilities in Mtwara Rural district and implemented for 18 months in 23 primary health facilities in Ruangwa district. The model brings together healthcare providers from different health facilities in interactive workshops by: applying QI methods to generate and test change ideas in their own facilities; using local data to monitor improvement and decision making; and health facility supervision visits by project and district mentors. The topics for improving childbirth were deliveries and partographs.

**Findings:**

Median monthly deliveries increased in 4 months from 38 (IQR 37-40) to 65 (IQR 53-71) in Mtwara Rural district, and in 17 months in Ruangwa district from 110 (IQR 103-125) to 161 (IQR 148-174). In Ruangwa health facilities, the women for whom partographs were used to monitor labour progress increased from 10 to 57 per cent in 17 months.

**Research limitations/implications:**

The time for QI innovation, testing and implementation phases was limited, and the study only looked at trends. The outcomes were limited to process rather than health outcome measures.

**Originality/value:**

Healthcare providers became confident in the QI method through engagement, generating and testing their own change ideas, and observing improvements. The findings suggest that implementing a QI initiative is feasible in rural, low-income settings.

## Introduction

Tanzania demographic and health survey (TDHS) reports from the past 20 years show that country is a developing country that suffers from high maternal and neonatal morbidity and mortality (Ministry of Health, Community Development, Gender, Elderly and Children (MoHCDGEC), Ministry of Health (MoH) (Zanzibar), National Bureau of Statistics (NBS), Office of the Chief Government Statistician (OCGS), and ICF, 2016). The TDHS 2004-2005 report revealed a countrywide maternal mortality ratio (MMR) estimated at 578 deaths per 100,000 live births and neonatal mortality rate at 32 deaths per 1,000 live births (National Bureau of Statistics (NBS) and ORC Macro, 2005). The southern Tanzania estimate for neonatal deaths was the highest in the country (47 deaths per 1,000 live births) (National Bureau of Statistics (NBS) and ORC Macro, 2005). Additionally, a study conducted in five southern Tanzania districts between 2004 and 2007 estimated MMR at 729 per 100,000 live births (Hanson, 2013). Many deaths are preventable through implementing effective and affordable interventions (Bhutta *et al.*, 2012; Khan *et al.*, 2006), including birth preparedness, labour monitoring (partograph) and immediate care for every neonate (Bhutta *et al.*, 2014; Lassi *et al.*, 2016; Ollerhead and Osrin, 2014). With more than 95 per cent of pregnant women attending antenatal care (ANC) at least once in the country and almost half delivering in health facilities (National Bureau of Statistics (NBS) and ICF Macro, 2011), there is a need to use high ANC attendances to offer quality services and help women to best prepare for their delivery, inform them about pregnancy-related complications and skilled delivery care (Magoma *et al.*, 2013; Magoma *et al.*, 2010). Improving institutional deliveries is a key strategy advocated to reduce maternal and neonatal deaths among the rural poor (Fogliati *et al.*, 2015). Tanzania has a vast primary health facility network serving most rural populations (Hanson *et al.*, 2013; Saronga *et al.*, 2014). However, most professional health workers are based in urban areas serving minority populations (National Bureau of Statistics (NBS) and ICF Macro, 2011). The gap created is compensated by informal task shifting; for example, medical attendants performing nursing duties and nurses performing clinical officer duties (Table I) (Manzi *et al.*, 2012), which has implications for healthcare service quality (Dawson *et al.*, 2014; Wiedenmayer *et al.*, 2015).

Evidence shows that poor quality facility-based care is a major contributing factor to elevated maternal and neonatal morbidity and mortality rates (Austin *et al.*, 2014). To improve healthcare service quality using existing personnel, several quality improvement (QI) approaches and models were introduced in Tanzania (Ministry of Health and Social Welfare, 2011) . Most QI programmes and studies focussed on hospitals (Anatole *et al.*, 2013; Berman *et al.*, 2012; Das *et al.*, 2014; Dumont *et al.*, 2013; Faye *et al.*, 2014; Ishijima *et al.*, 2014), and it was unclear if their benefits could be reproduced at dispensary and health centre levels. Different QI approaches, such as maternal and perinatal audits, collaborative improvement models, rapid improvement cycles, standard-based management and recognition, have been used in different studies to improve maternal and neonatal health outcomes (Dumont *et al.*, 2013; Kim *et al.*, 2013). A Malawi study used rapid QI cycles at health facilities to improve maternal, neonatal and perinatal mortality (Colbourn *et al.*, 2013). A Nicaraguan study also used rapid QI cycles to reduce hospital neonatal sepsis rates (Lopez *et al.*, 2013). Ifakara Health Institute (IHI) staff and their collaborators, through the Improving New-born Survival in Southern Tanzania (INSIST) project (Borghi *et al.*, 2013), together with the Ministry of Health and Social Welfare and Mtwara Rural and Ruangwa Council Health Management Teams (CHMT), explored the collaborative QI approach as a driver to improve healthcare processes during antenatal and childbirth care. We describe the developing and implementing a breakthrough series collaborative improvement model, which aimed to increase total health facility deliveries and women in labour for whom a partograph was used.

## Methods

### Study aim and objectives

The aim of this paper was to implement a QI intervention in health centres and dispensaries to improve maternal and neonatal health services in rural Southern Tanzania.

### Study setting and participants – 2009-2011

The intervention was piloted and implemented in dispensaries and health centres located in two southern Tanzania regions (Lindi and Mtwara). In the Mtwara region, the intervention was piloted in Mtwara Rural district (4/34 health facilities) and then implemented in Lindi region, Ruangwa district (23/24 health facilities). Mtwara Rural had population of 204,157 in 2002 (National Bureau of Statistics and Ministry of Planning, Economy and Empowerment, 2006). Ruangwa district had 124,009 inhabitants in 2002 (National Bureau of Statistics and Ministry of Planning, Economy and Empowerment, 2006). These health facilities have different healthcare providers working in reproductive and child health services (Table I), mainly clinical officers, nurses and medical attendants (Huicho *et al.*, 2008; Munga and Maestad, 2009).

### QI approach

**Figure 1:**
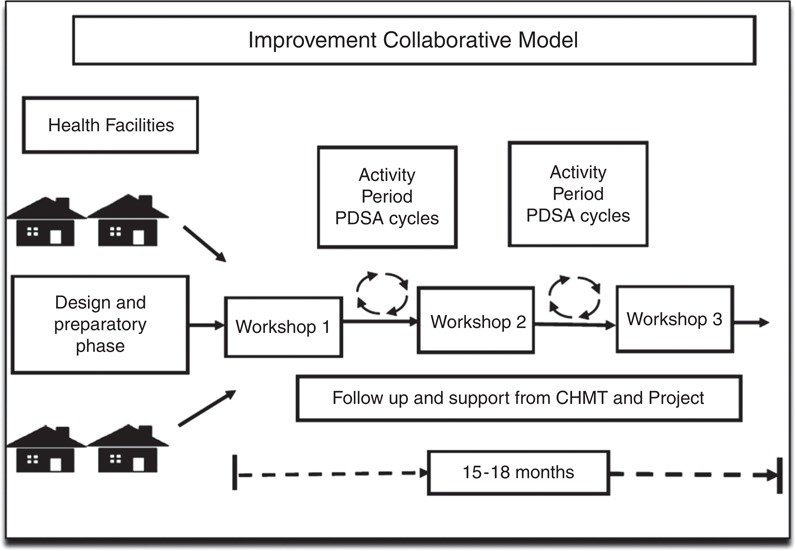
Improvement collaborative model

The breakthrough series collaborative QI model was used for improvement work (Figure 1) (Institute for Healthcare Improvement, 2003), which was chosen because it fosters rapid, data-driven improvements based on existing resources within a short time. This approach encouraged healthcare providers to develop aims, identify improvement that were shared amongst each other and tested through improvement cycles (plan-do-study-act cycles) (Taylor *et al.*, 2014). Throughout the process, data were collected and analysed to determine change effects, ensure data quality and build capacity to use data in decision making.

## The intervention

### Mtwara rural district

The southern Tanzania maternal and neonatal mortality figures were shared with the Mtwara Rural district CHMT, followed by discussions on what should be done to change the situation. The CHMT and project staff agreed to use the QI approach to improve outcomes. The district medical officer and reproductive child health (RCH) coordinator selected four health facilities based on their readiness to take part in QI activities. Additionally, a QI mentor was selected from the district healthcare workers to mentor and coach healthcare providers in health facilities that formed QI teams. At the initial workshop, QI team members, QI mentor, district managers and project staff discussed maternal and neonatal mortality and interventions. Pareto charts (Sokovic *et al.*, 2005) were used to prioritise potential causal issues; for example, most babies were born at home and care seeking behaviour for sick newborns was poor. Furthermore, healthcare providers pointed out that they did not properly counsel and advise women about childbirth and pregnancy danger signs. From this meeting, it was agreed to focus first on interventions that would encourage women to give birth in a health facility.

### Ruangwa district

A similar introductory process was used in Ruangwa district with CHMT and healthcare providers, and similar issues were identified. In Ruangwa, all 23 primary health facilities were involved in the intervention, and the QI teams adapted what was done in Mtwara rural district. They added a target to improve childbirth services using partographs to monitor and detect problems during labour because they noticed service gaps and wanted to improve service quality by providing appropriate care, detecting complications and referring complicated cases to the district hospital. The QI teams and the district managers met once during month three and five. In five iterative workshops attended by the QI teams, QI methods and how to apply them were taught by project staff and QI mentor. Maternal and new-born topics were revised, and QI teams shared their experiences, successes and challenges. After each workshop, the QI teams were visited every six weeks by project staff and the QI mentor, and were encouraged to test changes likely to bring improvement in their own facilities (Institute for Healthcare Improvement, 2003; USAID Health Care Improvement Project, 2008).

### Change topics

Mtwara Rural district QI teams chose to increase health facility deliveries by improving ANC counselling. The focus was to improve birth preparedness and knowledge about pregnancy danger signs (Table II). A pregnancy danger sign is defined as a symptom experienced by a woman that indicates a life-threatening condition in pregnancy that requires immediate action such as seeking help at the clinic or alerting a healthcare worker (Ministry of Health and Social Welfare, 2010). Staff perceived that there was a gap between what they (healthcare providers) should do and what was happening (Mushi *et al.*, 2010). Additionally, they thought that there was variability between provider performance in counselling pregnant women, which led them to not having enough knowledge about childbirth for them to make appropriate decisions. In Ruangwa district, the QI teams chose to focus on recording four partograph indicators to indicate completion: foetal heart rate (measured half-hourly); cervical dilatation (measured four-hourly); presenting part descent (measured four-hourly); and maternal blood pressure (BP) (measured half-hourly). If all four indicators were observed, then the partograph was considered complete.

### Execution

In Mtwara Rural district, IHI organised and led the project throughout, with CHMT, QI mentor and QI team support. The district QI mentor received full QI methods training lasting one day conducted by the QI project coordinator. Using that knowledge, the QI mentor facilitated three one-day QI workshops with the QI project coordinator, which were held between August and December 2009. Follow-up visits by the QI mentor, project coordinator and, sometimes, the District RCH coordinator occurred at least once every six weeks in between the workshops. The teams were encouraged to hold QI meetings at their health facilities to discuss how improvement work was going and how to improve further. However, this was rarely done owing to staff shortages, absenteeism and other competing responsibilities. To address the discontinuity caused by high staff turnover, it was agreed after every workshop that the QI team should give feedback to all staff at the health facility and should inform new staff about the QI activities. The QI intervention was rolled out to all primary healthcare facilities in Ruangwa district in 2010 using a similar approach.

Three improvements were implemented:

1. Mtwara Rural district QI teams aimed to increase median monthly health facility deliveries by 50 per cent within six months. Facility staff secure this improvement by testing and implementing the following changes: counsel every pregnant woman who attends ANC on birth preparedness and pregnancy danger signs, and documenting women receive the intervention; attend village meetings to raise awareness about health facility deliveries and new-born care in the community; conduct meetings with traditional birth attendants to raise facility deliveries and home delivery disadvantage awareness; and to foster friendly cooperation with healthcarers, so that women in labour would be referred to the facility.
2. Ruangwa district QI teams aimed to double the median monthly health facility deliveries within 17 months. The team adopted the change used in the Mtwara Rural collaborative, but added: health facility staff to invite husbands or mothers to accompany the ANC client to discuss birth preparedness and the plan for childbirth; and community volunteers to conduct home visits to pregnant women and give health education on facility delivery importance.

Partograph QI targets were implemented in Ruangwa district only. The aim was to increase total deliveries with a completed partograph from 10 per cent in February 2010 to 100 per cent by June 2011. Changes included:

1. conducting refresher training among healthcare providers during workshops and follow-up visits;
2. translating partographs from English to Swahili to make them understandable and consistent; and
3. using reminders for staff to conduct cheques at regular intervals, such as mobile phone alarms or prompts by relatives accompanying a woman in labour.

## Data collection and analysis

Health facility delivery data were collected monthly from the health management information system (HMIS) delivery registers. To verify HMIS data quality, we compared data from three sources: facility delivery register; partograph; and HMIS report. If there were discrepancies, then data from the delivery register were used. Partograph data were collected from partographs stored in the labour ward. Data were analysed using line graphs and Excel’s process control application, which helped to identify variation in improvement (Benneyan *et al.*, 2003; Taylor *et al.*, 2014; Timmerman *et al.*, 2010).

### Logistics and resources

In both districts, IHI covered meetings, stationery, refreshment and sitting-allowance costs. The sitting allowance was approximately 20 USD per participant. No other compensation was provided to the QI teams. Workshop participants were the QI teams, QI mentor and one district manager, and often the RCH coordinator. The IHI provided approximately six USD per day for the QI mentor during follow-up visits and transport for follow-up visits, although, occasionally, the district provided the vehicle and IHI provided fuel.

### Technical input

The IHI QI project coordinator (JJ) had a medical background and received QI advice from an experienced external QI advisor (CG). In June 2010, JJ undertook an IHI QI professional development programme.

## Ethics

This work was within the INSIST study that received ethical clearance through the National Institute of Medical Research Tanzania (NIMR/HQ/R.8c/Vol II/177), the IHI and LSHTM Institutional Review Board (LSHTM Reference No A358-5316). The study is registered on clinicaltrials.gov, number NCT01022788.

## Findings

### Pilot phase

**Figure 2:**
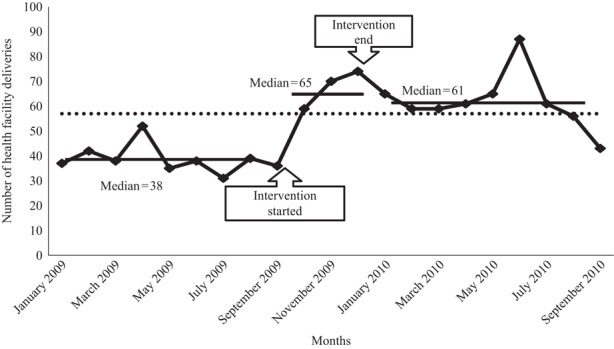
Median health facility deliveries per facility in Mtwara Rural collaborative (four facilities) from January 2009 to September 2010

**Figure 3:**
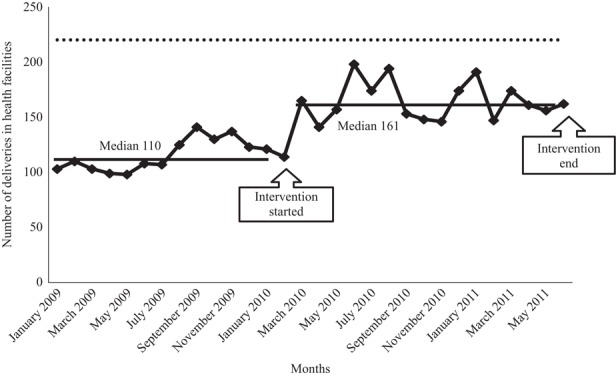
Median Health facility deliveries per facility in Ruangwa collaborative (23 facilities) from January 2009 to June 2011

**Figure 4:**
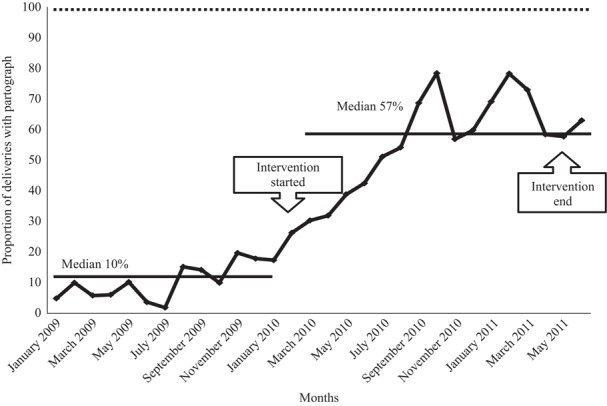
Median deliveries per facility in which partographs were completed in Ruangwa collaborative (23 facilities) from January 2009 to June 2011

From January to August 2009, the Mtwara Rural collaborative had a baseline median 38 (IQR 37-40) health facility deliveries per month (Figure 2). During the intervention phase (September to December 2009), the median was 65 (IQR 53-71) per month, a 71 per cent increase. This surpassed the health facility deliveries goal by 50 per cent (at December 2009). After December 2009, IHI staff ended the QI pilot in four Mtwara Rural health facilities. From January 2010 to September 2010, the improvement was sustained with a median 61 (IQR 59-65) health facility deliveries per month. However, median monthly facility deliveries declined from 56 in August to 43 in September, with no clear explanation from health facility staff (Figures 2-4).

### Implementation phase

The 23 Ruangwa collaborative health facilities had a baseline median 110 (IQR 103-125) deliveries per month from January 2009 to January 2010. During the 17 months over which the improvement work was undertaken, median deliveries per month in Ruangwa health facilities was 161 (IQR 148-174), a 46 per cent increase, missing the 100 per cent increase target by a wide margin (Figure 3). Between January 2009 and January 2010, the median deliveries at each facility with completed partographs was 10 per cent (IQR 6-15 per cent). Between February and June 2011, the median proportion was 57 per cent (IQR 42-69 per cent) – a 47 per cent increase, but missing the 100 per cent target by a wide margin.

## Discussion

The study demonstrated marked improvements in partograph use and in facility deliveries within rural settings using QI methods. Both pilot and implementation districts demonstrated improvement in their QI topics. The four pilot facilities in Mtwara Rural district maintained more than a 50 per cent improvement in health facility deliveries for 12 months. In Ruangwa district, facility deliveries increased by slightly less than 50 per cent and partograph use, despite missing the target, increased over five times from 10 to 57 per cent. These results are impressive considering that bringing women to deliver in health facilities requires a multi-sectorial approach involving many players (Gabrysch and Campbell, 2009). Using partograph for labour management in low-income settings is emphasised in a review by (Orhue *et al.*, 2012). Partographs detect labour complications and prompt healthcare staff to provide appropriate interventions to ensure feto-maternal wellbeing.

### Lessons learnt from the implementation process – technical support

For both acceptability and sustainability, local leaders needed to spearhead the intervention. However, our direct involvement in driving the improvement work delayed the district managers accepting the intervention. This experience supports the suggestion from Berman *et al.* (2012) that external assistance when developing QI approaches should focus on facilitation that supports local leadership to prioritise improvement projects and local health carer mentorship. Although we expected that the QI teams would hold monthly meetings autonomously, most were prompted by project staff. Webster *et al.* (2012) concluded that it is possible to increase access to HIV testing and treatment within a defined geographical area using QI methods to identify and spread successful, locally tested changes.

### Healthcare worker competence

Medical attendants formed most of the QI teams despite them not being considered competent to provide childbirth services according to the WHO skilled personnel definition (Spangler, 2012; World Health Organisation, 2004). They are not officially recognised as professional healthcare providers and hence they are not invited to relevant technical training, such as lifesaving skills, which is essential for childbirth services (Spangler, 2012). However, they are key care providers in rural facilities. Spangler noted that in rural Tanzania, many doctors, nurses, midwives and clinical officers do not possess competencies that qualify them as skilled owing to inconsistencies in pre-service training and regulation. Some birth attendants, who were not considered accredited professionals, practiced skilled care daily. During our study, medical attendants were actively involved without considering their educational level. This created a positive impact on their performance, but led to delays in understanding some technical issues; for example, most were learning how to complete partographs for the first time (Jaribu *et al.*, 2016).

### Continuing the intervention

While our results are promising, the extent to which the intervention has been sustained by the district managers is not known. It was made clear to the district managers up-front that the QI initiative would have external support from the project for 18 months, and thereafter they would need to take over implementation.

## Policy and practice implications

Although we observed clinical guidelines, such as focussed antenatal care, at all health facilities, their usage was limited. In some facilities, guidelines were unreachable. When we printed a one-page document for birth planning and pregnancy danger signs, healthcare staff complied well in using it with pregnant women. However, its sustainability after the project is unclear. The birth planning and pregnancy danger signs counselling guideline is one among many: we suggest that guidelines could be simplified in an electronic form that will be easily accessed and understandable (Lund *et al.*, 2016). An improving quality culture in the entire health system is needed to effectively use and profit from the fast growing QI initiatives introduced in the country (Atherton *et al.*, 1999). Tanzania is practicing service decentralisation at the district level, which provides a good platform for applying QI methods led by CHMTs, rather than by donor partners. One major opportunity is mandating district activities to be planned by themselves making the comprehensive council health plan, which prioritises and approves budgets and interventions to be implemented at the district level.

## Research implications

An observational study in ANC clinics is needed to ascertain barriers and facilitators for counselling services. Community involvement is another area to improve childbirth service quality. Partographs, as a tool, to improve labour services in health centres and dispensaries need further studies, taking into consideration different cadres that work at these facilities, especially how their skills and referrals to district hospitals can be improved.

### Limitations

Our study is limited by using trends over time, which could be due to changes in factors that are unrelated to the intervention. A comparison group would have improved the study’s internal validity. Additionally, HMIS data recording was inconsistent and data were stored poorly making them unreliable. The study’s QI innovation time was limited, and testing and implementation phases and the outcomes were limited to processes rather than health outcomes, such as mortality. While it is difficult to explain exactly what QI interventions led to improvement, we believe the improvements were likely attributable to increased staff involvement in identifying and testing their own solutions, associated with increased local data to set targets and monitor progress. This is similar to a South African study that looked at using QI to accelerate highly active antiretroviral treatment coverage (Webster *et al.*, 2012).

## Conclusion

In the fight to lower developing country maternal and neonatal deaths, we have shown that the QI approach can accelerate Tanzania’s existing evidence-based interventions in rural health facilities. Bearing in mind that there are many cadres with varying competencies working in such facilities, planning how to teach and support them to use QI tools and to understand technical issues, despite their qualifications, will be beneficial. Systematically involving the hierarchical health system management up-front might facilitate acceptance and sustainability.

## Figures and Tables

**Table I:** Health worker cadres, training, roles and responsibilities

Healthcare worker cadre	Pre-service training	Pre-service training	Roles and responsibilities
Clinical officer	Post-secondary school	2-4 years	Identify and treat common diseases and perform minor surgery Participate in the planning and implementation of basic health services Keep records of equipment and tools for offering services Keep records, prepare and provide implementation report Supervise performance of subordinate health staff
Nurse	Post-secondary school	2-3 years	Provide nursing care to all clients in the catchment area served by their facility Collect vital health statistics Direct and supervise subordinate nurses Provide counselling Provide services to patients at home Provide preventive services like vaccination and childbirth services
Medical attendant	Post-primary school	1 year	Clean equipment, wards and surrounding environment Help patients with disabilities to use toilet and shower Feed patients who need support Take patient samples to the laboratory for testing and monitor results Prepare materials for cleaning and close wounds Follow up patients’ medication requirements from drug store

**Source:** Human resource development circular No. 1 of 2009 service scheme for health worker cadres under the Ministry of Health and Social Welfare

**Table II:** Birth preparedness plan and pregnancy danger signs

Birth preparedness plan	Pregnancy danger signs
Reminding the woman of her expected date of delivery Identifying the place of birth Identifying a health facility with skilled personnel Identifying someone to take care of her family in her absence Preparing essential items necessary for a clean birth and warmth for both mother and baby such as cloths or clothes Preparing transport or funds and any other available resources in case of an emergency during labour Identifying decision-making family member to accompany the pregnant woman to the health facility Helping the pregnant woman to recognise the importance of delivering in a health facility	Lethargy, fatigue, breathlessness Vaginal bleeding during pregnancy Severe headache and/or blurred vision Loss of consciousness or convulsions Severe oedema (hands or face) Severe abdominal pain Early rupture of membranes before 37 weeks Leaking of amniotic fluid from the vagina Foul-smelling vaginal discharge Fever, chills, vomiting Decreased or absent foetal movement Contractions before 37 weeks (premature labour)

## References

[ref001] Anatole M., Magge H., Redditt V., Karamaga A., Niyonzima S., Drobac P., Mukherjee J.S., Ntaganira J., Nyirazinyoye L. and Hirschhorn L.R. (2013), “Nurse mentorship to improve the quality of health care delivery in rural Rwanda”, Nursing Outlook, Vol. 61 No. 3, pp. 137-144. 23164530 10.1016/j.outlook.2012.10.003

[ref002] Atherton F., Mbekem G. and Nyalusi I. (1999), “Project report: improving service quality: experience from the Tanzania family health project”, International Journal for Quality in Health Care, Vol. 11 No. 4, pp. 353-356. 10501606 10.1093/intqhc/11.4.353

[ref003] Austin A., Langer A., Salam R.A., Lassi Z.S., Das J.K. and Bhutta Z.A. (2014), “Approaches to improve the quality of maternal and newborn health care: an overview of the evidence”, Reproductive Health, Vol. 11 No. S2, p. 1. 25209614 10.1186/1742-4755-11-S2-S1 PMC4160919

[ref004] Benneyan J.C., Lloyd R.C. and Plsek P.E. (2003), “Statistical process control as a tool for research and healthcare improvement”, Quality and Safety in Healthcare, Vol. 12 No. 6, pp. 458-464. 10.1136/qhc.12.6.458 PMC1758030 14645763

[ref005] Berman J., Nkabane E.L., Malope S., Machai S., Jack B. and Bicknell W. (2012), “Developing a hospital quality improvement initiative in Lesotho”, International Journal of Health Care Quality Assurance, Vol. 27 No. 1, pp. 15-24. 10.1108/IJHCQA-01-2012-0010 24660514

[ref006] Bhutta Z.A., Cabral S., Chan C.-W. and Keenan W.J. (2012), “Reducing maternal, newborn, and infant mortality globally: an integrated action agenda”, International Journal of Gynecology and Obstetrics, Vol. 119 No. S1, pp. 13-17. 10.1016/j.ijgo.2012.04.001 22883919

[ref007] Bhutta Z.A., Das J.K., Bahl R., Lawn J.E., Salam R.A., Paul V.K., Sankar M.J., Blencowe H., Rizvi A., Chou V.B. and Walker N. (2014), “Can available interventions end preventable deaths in mothers, newborn babies, and stillbirths, and at what cost?”, The Lancet, Vol. 384 No. 9940, pp. 347-370. 10.1016/S0140-6736(14)60792-3 24853604

[ref008] Borghi J., Cousens S., Hamisi Y., Hanson C., Jaribu J., Manzi F., Marchant T., Mkumbo E., Mshinda H., Penfold S., Schellenberg D., Schellenberg J., Shamba D. and Tanner M. (2013), Study Protocol: Improving Newborn Survival in Rural Southern Tanzania: A Cluster-Randomised Trial to Evaluate the Impact of a Scaleable Package of Interventions at Community Level with Health System Strengthening, London School of Hygiene and Tropical Medicine, London.

[ref009] Colbourn T., Nambiar B., Bondo A., Makwenda C., Tsetekani E., Makonda-Ridley A., Msukwa M., Barker P., Kotagal U., Williams C., Davies R., Webb D., Flatman D., Lewycka S., Rosato M., Kachale F., Mwansambo C. and Costello A. (2013), “Effects of quality improvement in health facilities and community mobilization through women’s groups on maternal, neonatal and perinatal mortality in three districts of Malawi: MaiKhanda, a cluster randomized controlled effectiveness trial”, International Health, Vol. 5 No. 3, pp. 180-195. 24030269 10.1093/inthealth/iht011 PMC5102328

[ref010] Das J., Kumar R., Salam R., Lassi Z. and Bhutta Z. (2014), “Evidence from facility level inputs to improve quality of care for maternal and newborn health: interventions and findings”, Reproductive Health, Vol. 11 No. S2, p. 4. 25208539 10.1186/1742-4755-11-S2-S4 PMC4160922

[ref011] Dawson A.J., Buchan J., Duffield C., Homer C.S.E. and Wijewardena K. (2014), “Task shifting and sharing in maternal and reproductive health in low-income countries: a narrative synthesis of current evidence”, Health Policy and Planning, Vol. 29 No. 3, pp. 396-408. 23656700 10.1093/heapol/czt026

[ref012] Dumont A., Fournier P., Abrahamowicz M., Traoré M., Haddad S. and Fraser W.D. (2013), “Quality of care, risk management, and technology in obstetrics to reduce hospital-based maternal mortality in Senegal and Mali (QUARITE): a cluster-randomised trial”, The Lancet, Vol. 382 No. 9887, pp. 146-157. 10.1016/S0140-6736(13)60593-0 23721752

[ref013] Faye A., Dumont A., Ndiaye P. and Fournier P. (2014), “Development of an instrument to evaluate intrapartum care quality in Senegal: evaluation quality care”, International Journal for Quality in Health Care, Vol. 26 No. 2, pp. 184-189. 24585857 10.1093/intqhc/mzu018

[ref014] Fogliati P., Straneo M., Brogi C., Fantozzi P.L., Salim R.M., Msengi H.M., Azzimonti G. and Putoto G. (2015), “How can childbirth care for the rural poor be improved? A contribution from spatial modelling in rural Tanzania”, PLoS ONE, Vol. 10 No. 9, p. e0139460. 10.1371/journal.pone.0139460 PMC4589408 26422687

[ref015] Gabrysch S. and Campbell O.M. (2009), “Still too far to walk: literature review of the determinants of delivery service use”, BMC Pregnancy Childbirth, Vol. 9 No. 1, p. 34. 19671156 10.1186/1471-2393-9-34 PMC2744662

[ref016] Hanson C. (2013), The Epidemiology of Maternal Mortality in Southern Tanzania, unpublished PhD, London School of Hygiene and Tropical Medicine, London.

[ref017] Hanson C., Ronsmans C., Penfold S., Maokola W., Manzi F., Jaribu J., Mbaruku G., Mshinda H., Tanner M. and Schellenberg J. (2013), “Health system support for childbirth care in Southern Tanzania: results from a health facility census”, BMC Research Notes, Vol. 6 No. 1, p. 435. 24171904 10.1186/1756-0500-6-435 PMC4228478

[ref018] Huicho L., Scherpbier R.W., Nkowane A.M. and Victora C.G. (2008), “How much does quality of child care vary between health workers with differing durations of training? An observational multicountry study”, The Lancet, Vol. 372 No. 9642, pp. 910-916. 10.1016/S0140-6736(08)61401-4 18790314

[ref019] Institute for Healthcare Improvement (2003), The Breakthrough Series: IHI’s Collaborative Model for Achieving Breakthrough Improvement, Institute for Healthcare Improvement, Boston, MA.

[ref020] Ishijima H., Eliakimu E., Takahashi S. and Miyamoto N. (2014), “Factors influencing national rollout of quality improvement approaches to public hospitals in Tanzania”, Clinical Governance, Vol. 19 No. 2, pp. 137-152.

[ref021] Jaribu J., Penfold S., Manzi F., Schellenberg J. and Pfeiffer C. (2016), “Improving institutional childbirth services in rural Southern Tanzania: a qualitative study of healthcare workers’ perspective”, BMJ Open, Vol. 6 No. 9, p. e010317. 10.1136/bmjopen-2015-010317 PMC5051329 27660313

[ref022] Khan K.S., Wojdyla D., Say L., Gülmezoglu A.M. and Van Look P.F.A. (2006), “WHO analysis of causes of maternal death: a systematic review”, The Lancet, Vol. 367 No. 9516, pp. 1066-1074. 10.1016/S0140-6736(06)68397-9 16581405

[ref023] Kim Y.M., Chilila M., Shasulwe H., Banda J., Kanjipite W., Sarkar S., Bazant E., Hiner C., Tholandi M., Reinhardt S., Mulilo J.C. and Kols A. (2013), “Evaluation of a quality improvement intervention to prevent mother-to-child transmission of HIV (PMTCT) at Zambia defence force facilities”, BMC Health Services Research, Vol. 13 p. 345. 24011137 10.1186/1472-6963-13-345 PMC3852054

[ref024] Lassi Z.S., Middleton P.F., Bhutta Z.A. and Crowther C. (2016), “Strategies for improving health care seeking for maternal and newborn illnesses in low- and middle-income countries: a systematic review and meta-analysis”, Global Health Action, Vol. 9 No. 1, p. 31408. 10.3402/gha.v9.31408 PMC4864851 27171766

[ref025] Lopez S., Wong Y., Urbina L., Gomez I., Escobar F., Tinoco B. and Parrales A. (2013), “Quality in practice: preventing and managing neonatal sepsis in Nicaragua”, International Journal for Quality in Health Care, Vol. 25 No. 5, pp. 599-605. 23962992 10.1093/intqhc/mzt060

[ref026] Lund S., Boas I., Bedesa T., Fekede W., Nielsen H. and Sørensen B. (2016), “Association between the safe delivery app and quality of care and perinatal survival in Ethiopia: a randomized clinical trial”, The Journal of the American Medical Pediatrics, Vol. 170 No. 8, pp. 765-771. 10.1001/jamapediatrics.2016.0687 27322089

[ref027] Magoma M., Requejo J., Campbell O.M., Cousens S. and Filippi V. (2010), “High ANC coverage and low skilled attendance in a rural Tanzanian district: a case for implementing a birth plan intervention”, BMC Pregnancy and Childbirth, Vol. 10 No. 1, pp. 1-12. 20302625 10.1186/1471-2393-10-13 PMC2850322

[ref028] Magoma M., Requejo J., Campbell O., Cousens S., Merialdi M. and Filippi V. (2013), “The effectiveness of birth plans in increasing use of skilled care at delivery and postnatal care in rural Tanzania: a cluster randomised trial”, Tropical Medicine and International Health, Vol. 18 No. 4, pp. 435-443. 23383733 10.1111/tmi.12069

[ref029] Manzi F., Schellenberg J., Hutton G., Wyss K., Mbuya C., Shirima K., Mshinda H., Tanner M. and Schellenberg D. (2012), “Human resources for health care delivery in Tanzania: a multifaceted problem”, Human Resources for Health, Vol. 10 No. 1, p. 3. 22357353 10.1186/1478-4491-10-3 PMC3311084

[ref030] Ministry of Health and Social Welfare (2010), Huduma Muhimu za Afya ya Uzazi Wakati wa Ujauzito, Ministry of Health and Social Welfare, Dar es Salaam.

[ref031] Ministry of Health and Social Welfare (2011), The Tanzania Quality Improvement Framework in Health Care 2011-2016, Ministry of Health and Social Welfare, Dar es Salaam.

[ref032] Ministry of Health, Community Development, Gender, Elderly and Children (MoHCDGEC) [Tanzania, Mainland], Ministry of Health (MoH) (Zanzibar), National Bureau of Statistics (NBS), Office of the Chief Government Statistician (OCGS), and ICF (2016), “Tanzania demographic and health survey and Malaria indicator survey 2015-16”, MoHCDGEC, MoH, NBS, OCGS and ICF, Dar es Salaam, and Rockville, MD.

[ref033] Munga M. and Maestad O. (2009), “Measuring inequalities in the distribution of health workers: the case of Tanzania”, Human Resources for Health, Vol. 7 No. 1, p. 4. 19159443 10.1186/1478-4491-7-4 PMC2655278

[ref034] Mushi D., Mpembeni R. and Jahn A. (2010), “Effectiveness of community based safe motherhood promoters in improving the utilization of obstetric care: the case of Mtwara rural district in Tanzania”, BMC Pregnancy and Childbirth, Vol. 10 No. 1, p. 14. 20359341 10.1186/1471-2393-10-14 PMC2858713

[ref035] National Bureau of Statistics (NBS) and ICF Macro (2011), Tanzania Demographic and Health Survey 2010, National Bureau of Statistics and ICF Macro, Dar es Salaam.

[ref036] National Bureau of Statistics and Ministry of Planning, Economy and Empowerment (2006), Tanzania census 2002: Analytical Report, National Bureau of Statistics, Dar es Salaam.

[ref037] National Bureau of Statistics (NBS) and ORC Macro (2005), Tanzania Demographic and Health Survey 2004-05, National Bureau of Statistics and ORC Macro, Dar es Salaam.

[ref038] Ollerhead E. and Osrin D. (2014), “Barriers to and incentives for achieving partograph use in obstetric practice in low- and middle-income countries: a systematic review”, BMC Pregnancy Childbirth, Vol. 14 No. 1, p. 281. 25132124 10.1186/1471-2393-14-281 PMC4147181

[ref039] Orhue A., Aziken M. and Osemwenkha A. (2012), “Partograph as a tool for team work management of spontaneous labor”, Nigerian Journal of Clinical Practice, Vol. 15 No. 1, pp. 1-8. 22437079 10.4103/1119-3077.94087

[ref040] Saronga H., Duysburgh E., Massawe S., Dalaba M., Savadogo G., Tonchev P., Dong H., Sauerborn R. and Loukanova S. (2014), “Efficiency of antenatal care and childbirth services in selected primary health care facilities in rural Tanzania: a cross-sectional study”, BMC Health Services Research, Vol. 14 No. 1, p. 96. 24581003 10.1186/1472-6963-14-96 PMC3944798

[ref041] Sokovic M., Pavletic D. and Fakin S. (2005), “Application of six sigma methodology for process design”, Journal of Materials Processing Technology, Vol. 162-163 No. 1, pp. 777-783.

[ref042] Spangler S.A. (2012), “Assessing skilled birth attendants and emergency obstetric care in rural Tanzania: the inadequacy of using global standards and indicators to measure local realities”, Reproductive Health Matters, Vol. 20 No. 39, pp. 133-141. 22789091 10.1016/S0968-8080(12)39603-4

[ref043] Taylor M.J., McNicholas C., Nicolay C., Darzi A., Bell D. and Reed J.E. (2014), “Systematic review of the application of the plan-do-study-act method to improve quality in healthcare”, BMJ Quality and Safety, Vol. 23 No. 4, pp. 290-298. 10.1136/bmjqs-2013-001862 PMC3963536 24025320

[ref044] Timmerman T., Verrall T., Clatney L., Klomp H. and Teare G. (2010), “Taking a closer look: using statistical process control to identify patterns of improvement in a quality-improvement collaborative”, BMJ Quality and Safety, Vol. 19 No. 6, p. e19. 10.1136/qshc.2008.029025 20595718

[ref045] USAID Health Care Improvement Project (2008), The Improvement Collaborative: An Approach to Rapidly Improve Health Care and Scale up Quality Services, L.U. USAID Health Care Improvement Project: University Research Co., Bethesda, MD.

[ref046] Webster P.D., Sibanyoni M., Malekutu D., Mate K.S., Venter W.D., Barker P.M. and Moleko W. (2012), “Using quality improvement to accelerate highly active antiretroviral treatment coverage in South Africa”, BMJ Quality and Safety, Vol. 21 No. 4, pp. 315-324. 10.1136/bmjqs-2011-000381 PMC3311871 22438327

[ref047] Wiedenmayer K.A., Kapologwe N., Charles J., Chilunda F. and Mapunjo S. (2015), “The reality of task shifting in medicines management – a case study from Tanzania”, Journal of Pharmaceutical Policy and Practice, Vol. 8 No. 1, p. 13. 25893096 10.1186/s40545-015-0032-8 PMC4394399

[ref048] World Health Organization (2004), Making Pregnancy Safer: The Critical role of the Skilled Attendant: A Joint Statement by WHO, ICM and FIGO, WHO, Geneva.

